# NF-κB p65 iso5 Δ6/7 and p65 iso5 Δ10 isoforms drive immune-inflammatory remodeling in Fabry disease and *GLA* variants of uncertain significance

**DOI:** 10.3389/fimmu.2026.1922658

**Published:** 2026-10-06

**Authors:** Giuseppa Biddeci, Paolo Colomba, Gaetano Spinelli, Monia Anania, Irene Giacalone, Giovanni Duro, Margherita Stefania Rodolico, Tommaso Silvano Aronica, Francesco Di Blasi

**Affiliations:** 1 Institute for Biomedical Research and Innovation, National Research Council of Italy (CNR-IRIB), Palermo, Italy; 2 Institute for Biomedical Research and Innovation, National Research Council (CNR-IRIB), Section of Catania, Catania, Italy; 3 Complex Operative Unit of Clinical Pathology, Azienda di Rilievo Nazionale ed Alta Specializzazione (ARNAS) Civico Di Cristina e Benfratelli Hospitals, Palermo, Italy

**Keywords:** alternative splicing, Fabry disease, GLA variants of uncertain significance, immune-inflammatory remodeling, inflammatory signaling, NF-κB isoforms, p65 iso5 Δ10, p65 iso5 Δ6/7

## Abstract

**Introduction:**

Fabry disease (FD) is an X-linked lysosomal storage disorder in which chronic inflammation contributes to disease progression beyond substrate accumulation. Although nuclear factor kappa B (NF-κB) signaling has been implicated in FD pathogenesis, the contribution of alternative NF-κB splicing to immune-inflammatory remodeling remains unknown. Here, we investigated the recently identified NF-κB p65 iso5 splice variants p65 iso5 Δ6/7 and p65 iso5 Δ10 in FD and in individuals carrying GLA variants of uncertain significance (GVUS).

**Methods:**

We characterized p65 iso5 Δ6/7 and p65 iso5 Δ10 in peripheral blood mononuclear cells from 103 patients with FD, including classic and late-onset phenotypes, 30 GVUS subjects, and 30 healthy controls. Isoform expression was assessed at the mRNA and protein levels, and circulating inflammatory mediators were evaluated in selected groups.

**Results:**

Both isoforms were constitutively expressed under physiological conditions and were significantly upregulated at the mRNA level in FD, whereas p65 iso5 Δ6/7 was also increased at the protein level. A similar expression profile was observed in GVUS subjects despite preserved α-galactosidase A activity and normal Lyso-Gb3 levels. In carriers of the p.Ala143Thr (A143T) variant, increased isoform expression was associated with enhanced expression of pro-inflammatory cytokines and elevated circulating inflammatory mediators.

**Discussion:**

Together with the previously reported downregulation of p65 iso5, these findings indicate that selective NF-κB splice variant redistribution, rather than uniform pathway activation, characterizes immune-inflammatory remodeling in FD. Altered p65 isoforms expression may contribute to immune dysregulation and clinical heterogeneity in FD and *GLA* variants of uncertain significance.

## 1 Introduction

Fabry disease (FD; Online Mendelian Inheritance in Man [OMIM] #301500), is an X-linked lysosomal storage disorder caused by pathogenic variants in the *GLA* gene, which encodes the lysosomal enzyme α-galactosidase A (α-GalA) (1, 2). Deficiency of α-GalA leads to progressive accumulation of Gb3 (globotriaosylceramide) and its deacylated derivative, Lyso-Gb3 (globotriaosylsphingosine), in various cell types (3), resulting in a multisystemic disorder characterized by renal, cardiac, and cerebrovascular complications and reduced life expectancy (4). As an X-linked condition, the mutated *GLA* gene can be transmitted by affected males only to their daughters, while heterozygous females have a 50% chance of passing it to both their daughters and sons (2). Due to the phenomenon known as lyonization, which is the random inactivation of one X chromosome (XCI), females may present a wide phenotypic spectrum ranging from asymptomatic to severely affected, often with no direct correlation between genotype, enzyme activity, and clinical manifestation (2, 5–7). In male patients, diagnosis is typically established through enzymatic activity assays, whereas in females enzymatic testing may be unreliable due to X-chromosome lyonization; therefore, genetic analysis is required to confirm the diagnosis (8). A comprehensive diagnostic approach integrating clinical assessment, biochemical biomarkers, and molecular genetic analysis is essential for the accurate recognition and characterization of Fabry disease, particularly in patients with atypical presentations and in females (8, 9). Additional complexity arises from *GLA* variants of uncertain significance (GVUS), which may be associated with FD-like symptoms despite normal α-GalA activity and Lyso-Gb3 levels (10–12). Although enzyme replacement and substrate reduction therapies have improved clinical outcomes (13–15), accumulating evidence indicates that chronic inflammation plays a central role in FD pathogenesis, acting in parallel with substrate accumulation (16, 17). Glycosphingolipid overload disrupts lysosomal function, increases oxidative stress, impairs antigen presentation, and alters innate immune responses, ultimately promoting endothelial dysfunction and sustained inflammatory signaling (18–27).

Among these pathways, nuclear factor κB (NF-κB) signaling is crucial in regulating pro-inflammatory mediators such as interleukin-6 (IL-6), tumor necrosis factor-α (TNF-α), and interleukin-1 beta (IL-1β), which contribute to tissue remodeling, fibrosis, and organ dysfunction (18, 28–32). The activity of NF-κB is further integrated with other transcriptional regulatory networks involved in inflammatory responses, including pathways regulated by peroxisome proliferator-activated receptor-γ (PPAR-γ), highlighting the complex and context-dependent regulation of inflammatory signaling (33). Among the mechanisms that regulate the magnitude and specificity of inflammatory responses, alternative splicing has emerged as an important source of transcriptional diversity. Beyond canonical NF-κB activation, accumulating evidence indicates that alternative splicing of NF-κB components may contribute to the diversification of immune responses and inflammatory signaling programs (34–36). Recently, the involvement of NF-κB signaling in the inflammatory processes underlying Fabry disease has been highlighted, including the downregulation of p65 iso5, an alternative isoform of the NF-κB p65 subunit (30, 37). This alteration may reflect chronic inflammatory stimulation and lysosomal dysfunction-driven changes in NF-κB splice variant expression, potentially contributing to the impaired immune regulation observed in FD (21, 37, 38). These observations raise the possibility that chronic inflammatory stress in Fabry disease may be associated with selective redistribution of NF-κB splice variants rather than uniform activation of the pathway. Building on these hypotheses, we recently identified and molecularly characterized two new p65 iso5 splice variants, named p65 iso5 Δ6/7 and p65 iso5 Δ10, which exhibit distinct structural and biochemical properties compared with the previously identified p65 iso5 isoform. The p65 iso5 Δ6/7 variant arises from the skipping of exons 6 and 7, resulting in the loss of 106 amino acids (Ala188-Asp293) within the Rel Homology Domain (RHD), whereas p65 iso5 Δ10 originates from an intraexonic splicing event within exon 10, leading to the deletion of 103 amino acids (Ala406-Gln508) within the transactivation domain (TAD). Despite these structural differences, both isoforms retain the ability to bind dexamethasone, interact with the glucocorticoid receptor, and translocate to the nucleus independently of inhibitor of κB alpha (IκBα), suggesting unique regulatory functions in NF-κB-mediated inflammatory responses (38, 39). Although p65 iso5 Δ6/7 and p65 iso5 Δ10 have recently been identified and molecularly characterized, their expression profile and potential clinical significance in Fabry disease and in individuals carrying *GLA* variants of uncertain significance remain unexplored. In particular, it is currently unknown whether these alternatively spliced p65 isoforms are differentially expressed across distinct Fabry disease phenotypes or in individuals carrying *GLA* variants associated with clinical manifestations despite preserved biochemical parameters. To address this knowledge gap, we investigated the expression of p65 iso5 Δ6/7 and p65 iso5 Δ10 in peripheral blood mononuclear cells (PBMCs) from patients with classic and late-onset Fabry disease, individuals carrying *GLA* variants of uncertain significance (GVUS), and healthy controls. We further assessed whether changes in p65 iso5 Δ6/7 expression were accompanied by alterations in inflammatory cytokine profiles, with particular attention to individuals carrying the p.Ala143Thr (A143T) variant. Through this approach, we aimed to determine whether differential expression of these NF-κB splice variants is associated with the immune-inflammatory phenotype observed across Fabry disease and GVUS subjects and to explore their potential relevance as molecular signatures of disease heterogeneity.

## 2 Materials and methods

### 2.1 Study design and population

Genetic and biochemical analyses were performed at the Centre for Research and Diagnosis of Lysosomal Storage Disorders, CNR-IRIB, Palermo, Italy. The study cohort included 163 individuals: 58 patients with the classic Fabry disease phenotype, 45 with the late-onset phenotype, 30 subjects carrying *GLA* variants of uncertain significance (GVUS), and a control group of 30 healthy individuals (15 males and 15 females). Fabry disease diagnosis was established based on clinical evaluation, reduced α-galactosidase A (α-GalA) enzymatic activity, *GLA* genetic testing, and Lyso-Gb3 quantification. Following identification of a *GLA* variant by genetic analysis, its clinical significance was assessed based on the information available in databases such as HGMD and ClinVar. All participants were informed about the nature and purpose of the study, and written informed consent was obtained prior to inclusion.

### 2.2 Genetic analysis

Peripheral blood samples were collected in ethylenediaminetetraacetic acid (EDTA) tubes and spotted onto absorbent filter paper to generate dried blood spots (DBS). Genomic DNA was extracted from DBS using silica-coated magnetic particles on an automated robotic workstation for nucleic acid purification (Qiagen, Hilden, Germany). Mutations in the *GLA* gene were studied using Sanger sequencing performed by Eurofins Genomics (Germany). Eight primer pairs were designed to amplify eight target regions, covering the seven exons of the *GLA* gene, their flanking regulatory region, and the cryptic exon. Polymerase chain reaction (PCR) products were purified and sequenced using an automated DNA sequencer according to the manufacturer’s instructions.

### 2.3 α-Galactosidase A activity assay

α-galactosidase A (α-GalA) activity was measured using a dried blood filter paper (DBFP) assay based on the method described by Chamoles et al. (40) with minor modifications. A 10 μl blood spot corresponding to a 6 mm diameter disc, was placed in a 96-well plate suitable for fluorometric analysis and incubated for 18 h at 37 °C in a thermomixer (Eppendorf, Hamburg, Germany). The enzymatic reaction was stopped by adding 250 μL of 0.1 mol/L ethylenediamine buffer (pH 11.4). Background fluorescence was determined for each sample by performing a parallel reaction in the presence of 0.14 mmol/L 1-deoxygalactonojirimycin (DGJ; ≥98% purity; Cayman Chemical, 17179, Ann Arbor, MI, USA), a specific inhibitor of α-GalA, in citrate-phosphate buffer (pH 4.5). Background fluorescence was subtracted from total fluorescence to obtain specific enzymatic activity. Positive and negative controls, as well as a calibration curve generated using 4-methylumbelliferone, were included in each assay. Normal enzyme activity was defined as > 3.0 nmol/mL/h.

### 2.4 Lyso-Gb3 determination

Lyso-Gb3 concentrations in blood samples were quantified by tandem mass spectrometry (MS/MS), as previously described by Polo et al. (41). Lyso-Gb3 concentrations are expressed in nmol/L (normal range, 0.1-2.3 nmol/L).

### 2.5 Isolation of PBMCs from human samples

Peripheral blood samples were collected from 103 Fabry patients with classic and late-onset phenotype, 30 subjects with GVUS mutation and 30 healthy individuals. Samples were collected in Vacutainer tubes treated with EDTA as an anticoagulant. PBMCs from whole blood were isolated using Ficoll-Paque (Cytiva) density gradient centrifugation, according to the manufacturer’s protocol. Briefly, whole blood was diluted 1:2 with 1X phosphate-buffered saline (PBS1X) and gently layered over Ficoll-Paque. Samples were centrifuged at 970 × g for 30 minutes at room temperature with the brake off. The PBMCs layer was collected and washed twice with 1X PBS before downstream applications.

### 2.6 RNA extraction from PBMCs and RT-PCR

Total RNA was extracted from PBMCs using TRIzol reagent (Invitrogen) according to the manufacturer’s instructions. RNA concentration was determined using a NanoDrop One spectrophotometer (Thermo Scientific) by measuring absorbance at 260 nm. RNA purity was evaluated using the A260/A280 and A260/A230 ratios, and RNA integrity was assessed by agarose/formaldehyde gel electrophoresis. First-strand cDNA (starting from 2 µg of RNA) was synthesized using oligo(dT) primers, RNasin RNase inhibitor, Moloney murine leukemia virus (M-MLV) reverse transcriptase, and dNTPs (Promega), according to the manufacturer’s protocol.

### 2.7 Nested PCR

A dilution (1:20) of the reverse transcriptase reaction was used for nested PCR to specifically amplify the entire p65 iso5 mRNA product from PBMCs of Fabry disease patients, GVUS subjects and healthy controls. In the first PCR round, primers targeting exon -1 (Forward Ex-1: 5′-GTGACATCACCAAACTCCGCCGATC-3′) and the 3′ untranslated region (Reverse 3′UTR: 5′-AGAATCCGTAAGTGCTTTTGGAGG-3′) were used. For the second PCR round, 5 μL of a 1:40 dilution of the first-round PCR product was used as template. Internal primers targeting exon -1 (Forward Ex-1.1: 5′-TGAAATCCCCTAAAAACAAA-3′) and exon 10 (Reverse Ex10: 5′-TCTGGGGAGGGCAGGCGTCAC-3′) were employed. PCR products were separated on 1% agarose gels and visualized under UV illumination.

### 2.8 Real-time quantitative PCR

Quantitative real-time PCR (qPCR) was performed to assess the expression of NF-κB p65 iso5 isoforms and cytokines. Each 20 μL reaction contained: 10 μL ExcelTaq 2X Fast Q-PCR Master Mix (SYBR, ROX SMOBIO); 10 pmol of each primer; 20 ng cDNA; RNase-free water (Gibco). Primers for p65 iso5 Δ6/7 targeted exon 5 and exon 8, while primers for p65 iso5 Δ10 were designed to span the exon 10 splicing junction and the 3′ untranslated region (3′ UTR), as detailed in **Table 1**. All primer pairs were designed based on gene sequences obtained from the NCBI GenBank database and were validated for the absence of secondary structures, self-dimers, and for their amplification efficiency and specificity. All cDNA samples were analyzed in technical triplicates. qPCR was performed for 40 amplification cycles (95 °C for 20 s, 95 °C for 3 s, and 60 °C for 30 s). β-actin was used as the endogenous control. Non-template controls and reverse-transcription controls were included in each experiment. For qPCR, a StepOnePlus Real-Time PCR System (Applied Biosystems, Thermo Fisher) was used with 96-well PCR plates covered with MicroAmp Optical Adhesive Film (Applied Biosystems, Thermo Fisher). All the experiments were performed in triplicate and replicated twice. Relative expression levels were calculated using the comparative cycle threshold (Ct) method.

**Table 1:** Primer sequences used for the quantitative PCR analysis of p65 isoforms and inflammatory cytokines.

Target	Forward primer	Reverse primer
p65 iso5 Δ6/7	CCCATCTTTGACAATCACGATC	GCTGCGGGAAGGCACAGCAATGC
p65 iso5 Δ10	TATCAGCTCTGGCCCAGAGG	TCTGGGGAGGGCAGGCGTCAC
TNF-α	TCTTCTCGAACCCCGAGTGA	CCTCTGATGGCACCACCAG
IL-6	GTAGCCGCCCCACACAGACAGCC	GCCATCTTTGGAAGGTTC
IL-10	GTGATGCCCCAAGCTGA	CACGGCCTTGCTCTTGTTTT
IL-4	CCGTAACAGACATCTTTGCTGCC	GAGTGTCCTTCTCATGGTGGCT

The table reports the target transcripts and corresponding forward and reverse primer sequences used to assess the expression of p65 iso5 Δ6/7, p65 iso5 Δ10, TNF-α, IL-6, IL-10, and IL-4.

### 2.9 Cytokine analysis

Peripheral blood samples were centrifuged at 970 × g for 30 min at 4 °C to isolate plasma, which was stored at -80 °C until analysis. Cytokine concentrations (IFN-γ, IL-2, IL-4, IL-8, IL-10, IL-12, and adiponectin) were measured through the chemiluminescence (EIA) method on Cobas 8000 analyzer (Roche Diagnostics GmbH), according to the manufacturer’s instructions.

### 2.10 Protein sample preparation

Following PBMCs isolation, cells were lysed in TRIzol reagent, and proteins were extracted from the organic phase according to the manufacturer’s protocol, with minor modifications. Protein pellets were solubilized in lysis buffer (7 M urea, 2 M thiourea, 30 mM CHAPS, 2% Triton X-100, 39 mM Tris pH 8.8, 65 mM dithiothreitol (DTT), 1 mM Na_3_VO_4_, 1% protease inhibitor cocktail). Samples were incubated at 50 °C for 20 min and centrifuged at 10,000 × g for 10 min at 15 °C. Supernatants containing total protein were collected and stored at -80 °C. Protein concentration was determined using the Bradford assay (Bio-Rad).

### 2.11 Western blot analysis

Proteins were loaded in equal amounts (30 µg per lane) and separated by sodium dodecyl sulfate-polyacrylamide gel electrophoresis (SDS-PAGE) on 10% polyacrylamide gels and transferred onto 0.45 μm nitrocellulose membranes (GE Healthcare) at 50 V overnight at 4 °C in Tris/glycine/20% methanol transfer buffer under agitation. Membranes were blocked in 5% BSA in tris-buffered saline (TBS) buffer with gentle agitation for 30’ at room temperature. The membranes were incubated at room temperature in a fresh solution of 5% bovine serum albumin (BSA) in tris-buffered saline with Tween-20 (TBS-T) with the primary antibodies: anti-NF-κB p65 (D14E12, Cell Signaling; 1:5000) and anti β-Actin (sc-47778, Santa Cruz; 1:10000) for 2 h at room temperature. After incubation with primary antibodies, membranes were washed twice with TBS-T (7 min. each) and re-incubated with Alexa Fluor 680 goat anti-rabbit IgG (A21109, Invitrogen; 1:10:000) and Alexa Fluor 790 goat anti-mouse IgG (A11357, Invitrogen; 1:10:000) in 5% BSA in TBS-T for 1h at room temperature. The membranes were washed three times with TBS-T (5 min. each), and once with TBS (5 min.). Signals were detected using an Odyssey Infrared Imaging System (LI-COR Biosciences). Cos-1 cells transfected with expression vectors encoding p65 iso5 Δ6/7 were used as positive controls. The band intensities were quantified by densitometric analysis using ImageJ software. Expression levels were normalized to the housekeeping protein and reported as fold change relative to the mean of the control group.

### 2.12 Statistical analysis

Statistical analyses were performed using GraphPad Prism software (version 7). Data distribution was assessed before statistical analysis, and parametric tests were used for normally distributed data. Comparisons between two independent groups were performed using an unpaired two-tailed Student’s t-test. A p-value < 0.05 was considered statistically significant.

## 3 Results

### 3.1 Study population and phenotypic distribution

A total of 163 individuals were included in the study: 103 patients with a confirmed diagnosis of Fabry disease (FD), 30 subjects with *GLA* variants of uncertain significance, and 30 healthy controls (**Figure 1A**). Based on genotype, clinical presentation and data available from public databases (such as HGMD and ClinVar), FD patients were classified as having a classic (*n* = 58) or late-onset (*n* = 45) phenotype (**Figure 1B**). The classic FD subgroup included 24 males (41.38%) and 34 females (58.62%), whereas the late-onset subgroup included 19 males (42.22%) and 26 females (57.78%). The most frequent mutations in the classic subgroup were p.Gly85Asp (G85D, *n* = 12), p.Arg220Ter (R220X, *n* = 5), p.Gly261Cys (G261C, *n* = 5), p.Arg356Trp (R356W, *n* = 4), and 718_719delAA (*n* = 4). In the late-onset subgroup, the most frequent variants were p.Asn215Ser (N215S, *n* = 11), p.Arg301Gly (R301G, *n* = 10), p.Gly395Ala (G395A, *n* = 9), p.Phe113Leu (F113L, *n* = 8), and p.Met51Ile (M51I, *n* = 7). The complete genetic characteristics of the FD cohort are reported in **Table 2**. Healthy controls exhibited normal α-GalA enzymatic activity and Lyso-Gb3 levels, with no significant differences between males and females (**Table 3**). As expected, patients with classic FD showed markedly reduced α-GalA activity and increased Lyso-Gb3 levels. Females retained higher residual α-GalA activity than males, whereas males showed higher Lyso-Gb3 concentrations. Detailed genotype- and sex-specific biochemical data for the most frequent classic FD variants are reported in **Table 4**. Late-onset patients exhibited relatively preserved enzymatic activity and lower substrate accumulation, consistent with the partial α-GalA deficiency linked to these variants. Sex-stratified analysis showed that males, even within this subgroup, had reduced α-GalA activity and elevated Lyso-Gb3 levels (**Table 5**). These findings align with previous reports indicating that late-onset mutations lead to partial enzyme deficiency and a delayed clinical presentation, typically involving cardiac or renal systems during adulthood (42, 43). The age and sex distribution of the study groups is shown in **Figure 1C**. Age is a recognized modulator of immune function and systemic inflammatory tone, a phenomenon commonly referred to as “*inflammaging*” (44, 45). The comparable age distribution across groups reduces the likelihood that age-related inflammatory changes substantially influenced NF-κB isoform expression (**Figure 1C**). Within the classic subgroup, females were older on average, reflecting the delayed and variable clinical expression associated with X-chromosome inactivation, whereas hemizygous males typically exhibited earlier and more severe manifestations (46–48). Late-onset males were older than classic males, consistent with the natural history of variants associated with partial enzymatic deficiency and delayed organ involvement. The subjects included in the study were either symptomatic, presenting with clinical features commonly associated with FD (e.g., angiokeratomas, acroparesthesia, intolerance to heat or cold, ventricular hypertrophy, and renal dysfunction) or individuals evaluated through family screening due to a confirmed diagnosis. Neurological involvement was the most prevalent feature in classic FD, particularly among males, whereas cardiac manifestations predominated in the late-onset phenotype. The genetic and demographic characteristics of individuals carrying *GLA* variants of uncertain significance are summarized in **Table 6**. The classification of these variants was established according to public databases (such as HGMD and ClinVar). Among the 30 GVUS subjects, A143T was the most prevalent variant (*n* = 18), followed by p.Ser126Gly (S126G, *n* = 9), with other variants detected in single cases. Despite normal α-GalA enzymatic activity and plasma Lyso-Gb3 levels (**Table 7**), a substantial proportion of GVUS subjects presented clinical manifestations commonly associated with FD. This clinical-biochemical discrepancy indicates that the presence of clinical manifestations is not always accompanied by measurable abnormalities in conventional biochemical markers. The distribution of the major clinical manifestations across the study groups is shown in **Table 8**. These findings provide a clinical framework for interpreting the molecular alterations described in the subsequent sections.

**Figure 1:**
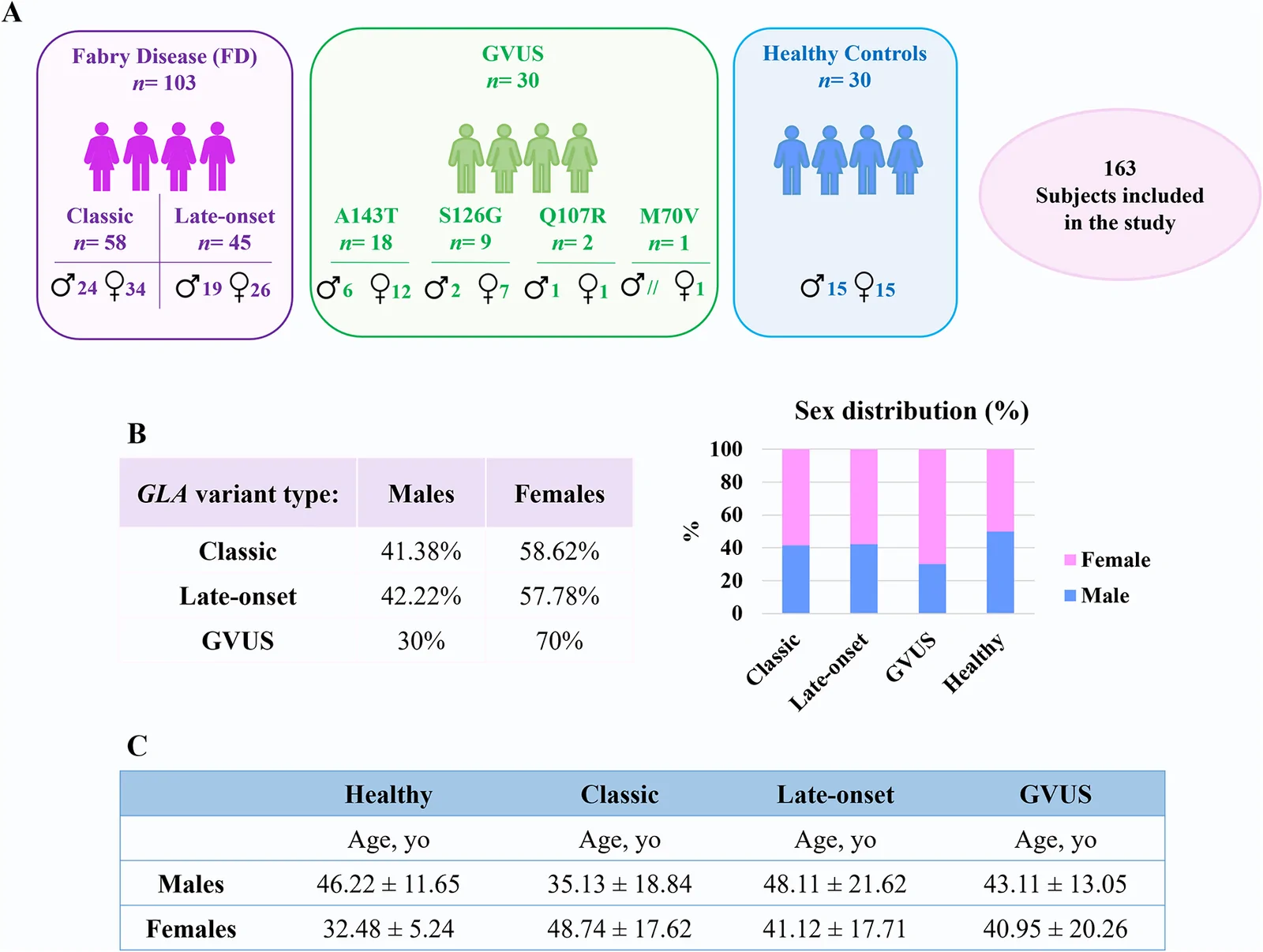
Demographic and clinical characteristics of the study cohort. **(A)** Overview of the study population, including 103 patients with Fabry disease (FD), 30 individuals carrying *GLA* variants of uncertain significance (GVUS), and 30 healthy controls, for a total of 163 subjects. FD patients were further classified into classic (*n* = 58) and late-onset (*n* = 45) phenotypes. The GVUS group included carriers of the p.Ala143Thr (A143T, *n* = 18), p.Ser126Gly (S126G, *n* = 9), p.Gln107Arg (Q107R, *n* = 2), and p.Met70Val (M70V, *n* = 1) variants. Sex distribution within each subgroup is indicated. **(B)** Sex distribution across the study groups expressed as percentages. **(C)** Mean age (± SD) of male and female participants in the healthy control, classic FD, late-onset FD, and GVUS groups.

**Table 2:** Demographic and genetic characteristics of patients with classic and late-onset Fabry disease.

*FD*	Patients (*n*)	Males (*n*)	Females (*n*)	Most frequent mutations (*n*)	Rare single variants (*n* = 1)
*Classic*	58	24	34		
				G85D (12), R220X (5), G261C (5), R356W (5), 718_719delAA (4), G361R (3), L32P (3), G183S (3),	1094dupA, L186V, L428X, G260E, R301P, c.114delG, L414C85*3, IVS2-2A>G, G360S, Del exon7, c.317-327del11, M187V, Y207X, IVS5-3delAC, IVS5-1G>C, R363H, W204X, Del exon2
*Late-onset*	45	19	26		
				N215S (11), R301G (10), G395A (9), F113L (8), M51I (7)	/

The table summarizes the number and sex distribution of patients, the most frequent *GLA* variants, and rare single variants in the classic and late-onset Fabry disease subgroups. Variant nomenclature follows the internationally recognized Human Genome Variation Society (HGVS) recommendations. Amino acid variants are shown in the table using the conventional one-letter amino acid code; their corresponding HGVS protein-level nomenclature is as follows: G85D (p.Gly85Asp), R220X (p.Arg220Ter), G261C (p.Gly261Cys), R356W (p.Arg356Trp), G361R (p.Gly361Arg), L32P (p.Leu32Pro), G183S (p.Gly183Ser), L186V (p.Leu186Val), L428X (p.Leu428Ter), G260E (p.Gly260Glu), R301P (p.Arg301Pro), G360S (p.Gly360Ser), M187V (p.Met187Val), Y207X (p.Tyr207Ter), R363H (p.Arg363His), W204X (p.Trp204Ter), N215S (p.Asn215Ser), R301G (p.Arg301Gly), G395A (p.Gly395Ala), F113L (p.Phe113Leu), and M51I (p.Met51Ile).

**Table 3:** Biochemical characteristics of healthy control subjects stratified by sex.

*Healthy subjects*	Average α-GalA activity mean (min - max)	Average Lyso-Gb3 mean (min - max)
*Males (n = 15)*	7.75 (6.8 - 9.65)	1.13 (0.45 - 1.38)
*Females (n = 15)*	7.14 (6.85 - 8.25)	0.98 (0.45 - 1.18)

The table reports α-GalA enzymatic activity and plasma Lyso-Gb3 concentrations as mean (min - max) in male and female healthy controls. α-GalA activity is expressed in nmol/mL/h (normal values >3 nmol/mL/h), whereas Lyso-Gb3 concentrations are expressed in nmol/L (normal range, 0.1-2.3 nmol/L).

**Table 4:** Biochemical characteristics of patients with classic Fabry disease according to the most frequent *GLA* mutations.

*Mutation*	Average α-GalA activity mean (min - max)	Average Lyso-Gb3 mean (min - max)
*G85D*		
*Males (n = 3)*	0.1 (0 - 0.2)	64.51 (60.69 - 71.38)
*Females (n = 9)*	8.71 (6.2 - 13.2)	6.87 (2.93 - 9.42)
*G261C*		
*Males (n = 2)*	1.3 (0.4 - 2.2)	12.7 (10.3 - 15.1)
*Females (n = 3)*	2.4 (1.8 - 2.8)	5.7 (3.82 - 7)
*R220X*		
*Males (n = 2)*	0.45 (0.1 - 0.8)	89.23 (40.97 - 137.48)
*Females (n = 3)*	4.2 (0.9 - 8.5)	14.47 (5.22 - 24.18)
*R356W*		
*Males (n = 2)*	0.4 (0.3 - 0.5)	9.16 (8.50 - 9.82)
*Females (n = 3)*	9.65 (8.2 - 11.1)	1.91 (1.2 - 2.62)
*718-719delAA*		
*Males (n = 2)*	0.27 (0.2 - 0.34)	40.72 (35.5 - 45.94)
*Females (n = 2)*	6.3 (4.1 - 8.5)	6.55 (4.45 - 8.65)
*G361R*		
*Males (n = 1)*	0.1 (0.1 - 0.1)	21.25 (21.25 - 21.25)
*Females (n = 2)*	6.1 (4.1 - 8.1)	7.32 (7.13 - 7.51)
*L32P*		
*Males (n = 1)*	0.4 (0.4 - 0.4)	92.25 (92.25 - 92.25)
*Females (n = 2)*	11.65 (8.3 - 15)	5.03 (4.84 - 5.22)
*G183S*		
*Males (n = 2)*	0.75 (0.1 - 1.4)	72.53 (65.02 - 80.03)
*Females (n = 1)*	1.2 (1.2 - 1.2)	19.59 (19.59 - 19.59)

The table reports sex-stratified α-GalA enzymatic activity and plasma Lyso-Gb3 concentrations as mean (min - max) for each mutation. α-GalA activity is expressed in nmol/mL/h (normal values >3 nmol/mL/h), whereas Lyso-Gb3 concentrations are expressed in nmol/L (normal range, 0.1-2.3 nmol/L).

**Table 5:** Biochemical characteristics of patients with late-onset Fabry disease according to the most frequent *GLA* mutations.

*Mutation*	Average α-GalA activity mean (min - max)	Average Lyso-Gb3 mean (min - max)
*N215S*		
*Males (n = 6)*	2.47 (0.7 - 4.7)	6.32 (4.45 - 9.29)
*Females (n = 5)*	5.65 (2.5 - 15.4)	1.78 (1.15 - 2.54)
*R301G*		
*Males (n = 6)*	1.46 (1 - 2.5)	9.85 (6.74 - 11.45)
*Females (n = 4)*	6.06 (2.8 - 12.05)	1.75 (1.02 - 2.93)
*G395A*		
*Males (n = 4)*	2.3 (1.8 - 2.8)	3.61 (3.42 - 3.79)
*Females (n = 5)*	6.03 (0.1 - 13.60)	1.48 (1.27 - 1.78)
*F113L*		
*Males (n = 2)*	2.10 (1.9 - 2.3)	5.35 (4.96 - 5.73)
*Females (n = 6)*	11.17 (3 - 26.6)	1.59 (1.4 - 2.16)
*M51I*		
*Males (n = 1)*	2.6 (2.6 - 2.6)	3.93 (3.93 - 3.93)
*Females (n = 6)*	10.52 (6.5 - 13.9)	1.03 (1.02 - 1.15)

The table reports sex-stratified α-GalA enzymatic activity and plasma Lyso-Gb3 concentrations as mean (min - max) for each mutation. α-GalA activity is expressed in nmol/mL/h (normal values >3 nmol/mL/h), whereas Lyso-Gb3 concentrations are expressed in nmol/L (normal range, 0.1-2.3 nmol/L).

**Table 6:** Demographic and genetic characteristics of subjects carrying *GLA* variants of uncertain significance (GVUS).

	Patients (*n*)	Males (*n*)	Females (*n*)	Most frequent mutations (*n*)	Rare single variants (*n* = 1)
*GVUS*	30	9	21		
				A143T (18), S126G (9), Q107R (2)	M70V

The table summarizes the number and sex distribution of GVUS subjects and the frequency of the most common and rare *GLA* variants identified in this subgroup. Variant nomenclature follows the internationally recognized Human Genome Variation Society (HGVS) recommendations. Amino acid variants are shown in the table using the conventional one-letter amino acid code; their corresponding HGVS protein-level nomenclature is as follows: A143T (p.Ala143Thr), S126G (p.Ser126Gly), Q107R (p.Gln107Arg), and M70V (p.Met70Val).

**Table 7:** Biochemical characteristics of subjects carrying *GLA* variants of uncertain significance (GVUS), stratified by variant and sex.

*Mutation*	Average α-GalA activity mean (min - max)	Average Lyso-Gb3 mean (min - max)
*A143T*		
*Males (n = 6)*	8.07 (4 - 12)	1.89 (1.53 - 2.67)
*Females (n = 12)*	9.36 (4.5 - 23.3)	1.25 (0.89 - 2.04)
*S126G*		
*Males (n = 2)*	20.35 (20 - 20.7)	1.66 (1.41 - 1.91)
*Females (n = 7)*	18.77 (11.4 - 31.4)	1.47 (0.76 - 2.23)
*Q107R*		
*Males (n = 1)*	18.1 (18.1 - 18.1)	1.53 (1.53 - 1.53)
*Females (n = 1)*	14.8 (14.8 - 14.8)	1.65 (1.65 - 1.65)

The table reports α-GalA enzymatic activity and plasma Lyso-Gb3 concentrations as mean (min - max) for the different GVUS variants. α-GalA activity is expressed in nmol/mL/h (normal values >3 nmol/mL/h), whereas Lyso-Gb3 concentrations are expressed in nmol/L (normal range, 0.1-2.3 nmol/L).

**Table 8:** Clinical manifestations in classic, late-onset, and GVUS Fabry disease subgroups stratified by sex.

*Classic (n = 58)*	Symptoms	Males (*n* = 24)	Females (*n* = 34)
	Cardiac manifestations	10 (41.7%)	8 (23.5%)
	Renal manifestations	12 (50%)	19 (55.9%)
	Neurological manifestations	16 (66.7%)	15 (44.1%)
*Late-onset (n = 45)*	Symptoms	Males (*n* = 19)	Females (*n* = 26)
	Cardiac manifestations	11 (57.9%)	15 (57.7%)
	Renal manifestations	8 (42.1%)	7 (26.9%)
	Neurological manifestations	8 (42.1%)	13 (50.4%)
*GVUS (n = 30)*	Symptoms	Males (*n* = 9)	Females (*n* = 21)
	Cardiac manifestations	1 (11.1%)	5 (23.8%)
	Renal manifestations	4 (44.4%)	16 (76.2%)
	Neurological manifestations	7 (77.8%)	12 (57.1%)

The table summarizes the frequency of cardiac, renal, and neurological manifestations in each phenotypic subgroup. Data are presented as number and percentage of male and female subjects within each subgroup.

### 3.2 Identification of p65 iso5 Δ6/7 and p65 iso5 Δ10 in Fabry PBMCs

Given the clinical heterogeneity of FD and the incomplete genotype-phenotype correlation, additional regulatory mechanisms may contribute to disease variability and progression (43, 49). In our previous studies, we identified alternative splicing isoforms of the NF-κB p65 subunit, including p65 iso5, p65 iso5 Δ6/7, and p65 iso5 Δ10, whose expression is modulated under inflammatory conditions and may contribute to the regulation of NF-κB dependent transcriptional responses (37–39). Considering the persistent inflammatory milieu associated with Fabry disease, driven by glycosphingolipid accumulation and lysosomal dysfunction (17, 21, 30), we investigated the presence of p65 iso5 Δ6/7 and p65 iso5 Δ10 transcripts in PBMCs from FD patients, GVUS subjects, and healthy controls. To amplify full-length transcripts, nested PCR was performed using primers targeting exon -1 and exon 10, located within the 5′ and 3′ untranslated regions (UTRs), respectively (**Figure 2A**). This strategy allows amplification of the full-length p65 iso5 transcript, generating the expected 1694 bp amplicon, as well as lower-molecular-weight products of approximately 1,400 bp corresponding to alternatively spliced transcripts. The expected amplicon sizes for splice variants were 1376 bp for p65 iso5 Δ6/7 and 1385 bp for p65 iso5 Δ10. Agarose gel analysis of the nested PCR products revealed the expected amplification pattern in representative samples from healthy controls, classic FD patients, late-onset FD patients, and GVUS subjects (**Figure 2B**).

**Figure 2:**
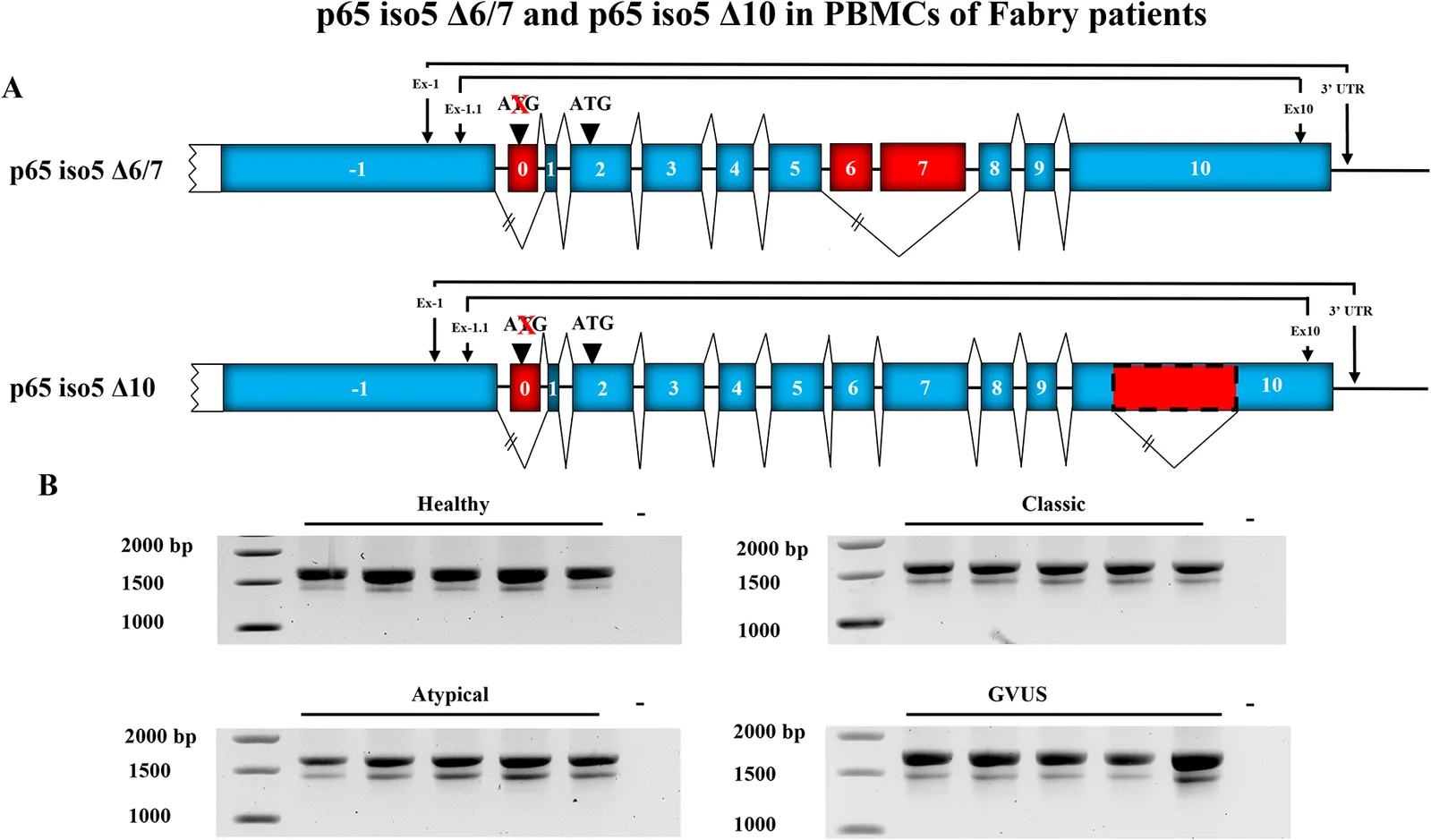
Identification of alternatively spliced p65 iso5 Δ6/7 and p65 iso5 Δ10 transcripts in PBMCs by nested PCR. **(A)** Schematic representation of the nested PCR strategy used to amplify full-length p65 iso5 Δ6/7 and p65 iso5 Δ10 transcripts. Primers annealed to exon -1 and exon 10 within the 5′ and 3′ untranslated regions (UTRs), respectively. The missing exons are highlighted in red. This strategy allows amplification of the full-length p65 iso5 transcript (1694 bp) as well as shorter alternatively spliced transcripts. The expected amplicon sizes are 1376 bp for p65 iso5 Δ6/7 and 1385 bp for p65 iso5 Δ10. **(B)** Representative agarose gel electrophoresis showing amplification of the p65 iso5 (1694 bp) and the two alternatively spliced isoforms in PBMCs from healthy controls, classic FD patients, late-onset FD patients, and GVUS subjects.

Isoform-specific PCR targeting unique exon-exon junctions confirmed selective amplification of each variant (**Figures 3A, B**). Sequencing of cloned products validated the identity of the transcripts and the presence of exon -1. Taken together, these findings demonstrate that p65 iso5 Δ6/7 and p65 iso5 Δ10 are detectable under physiological conditions and are not restricted to pathological states. Their relevance in Fabry disease is therefore more likely attributable to quantitative differences in expression rather than their exclusive presence, potentially reflecting dynamic regulation in response to inflammatory signaling.

**Figure 3:**
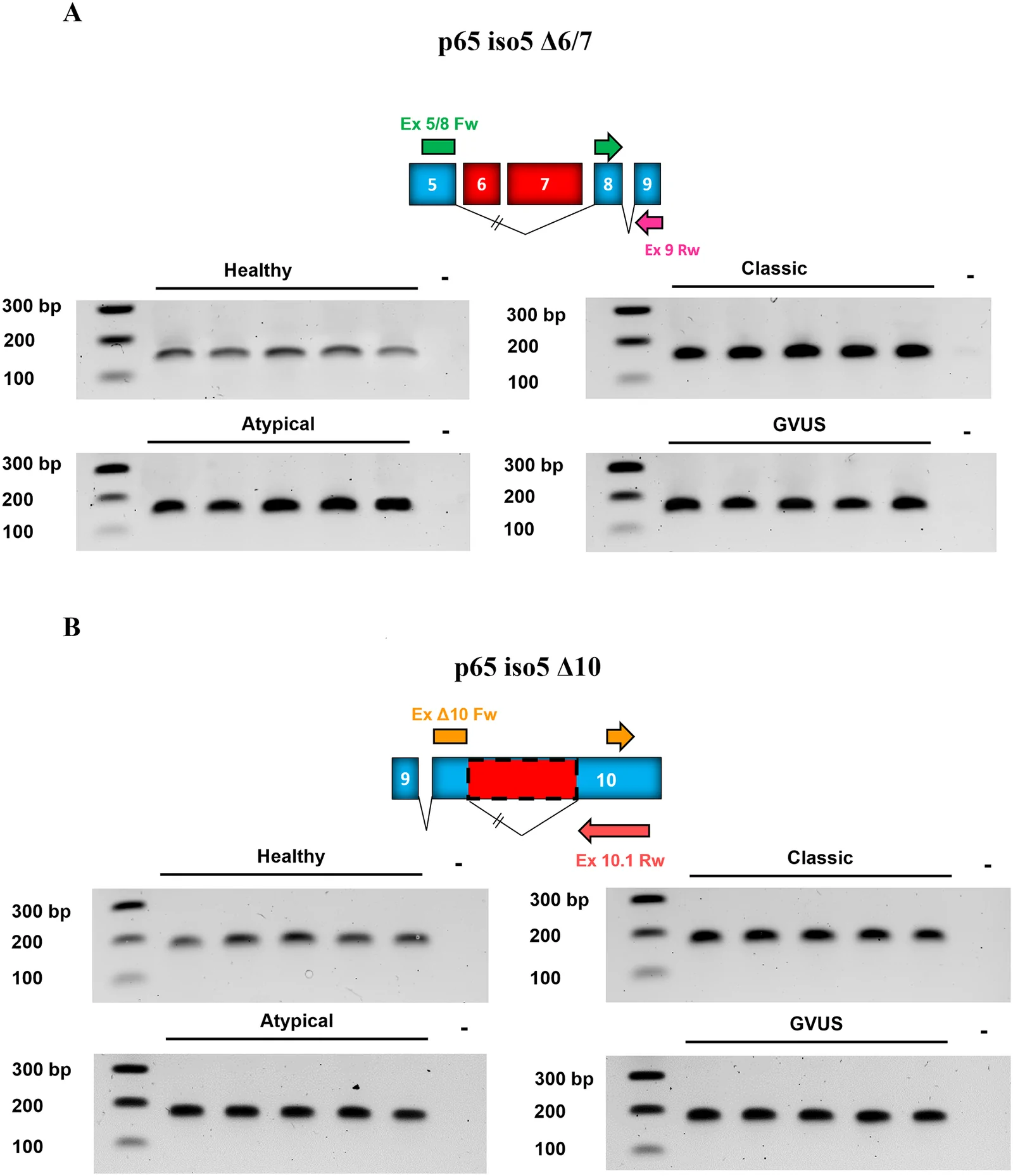
Validation of isoform-specific PCR assays for p65 iso5 Δ6/7 and p65 iso5 Δ10. **(A)** Schematic representation of the primer design targeting the unique exon-exon junction of p65 iso5 Δ6/7 and representative agarose gel electrophoresis demonstrating specific amplification of the Δ6/7 isoform. **(B)** Schematic representation of the primer design used for selective amplification of p65 iso5 Δ10 and representative agarose gel electrophoresis confirming isoform-specific amplification.

### 3.3 mRNA expression of p65 iso5 Δ6/7 and p65 iso5 Δ10 in Fabry PBMCs

To determine whether the expression of p65 iso5 Δ6/7 and p65 iso5 Δ10 is altered in FD, we quantified their mRNA levels in 103 FD patients with classic and late-onset phenotypes and compared them with healthy controls. Quantitative real-time PCR analysis revealed a significant upregulation of both isoforms in FD patients. Expression of p65 iso5 Δ6/7 was markedly increased in classic FD patients (**Figure 4A**), with similar trends observed in both males and females. A comparable pattern was detected in late-onset patients (**Figure 4B**), indicating that upregulation of this isoform is not restricted to severe enzymatic deficiency. Expression of p65 iso5 Δ10 was likewise significantly elevated in classic (**Figure 4C**) and late-onset FD patients (**Figure 4D**). These results demonstrate that alternative NF-κB p65 splicing is consistently altered across FD phenotypes, supporting the hypothesis that chronic inflammatory signaling contributes to isoform-specific remodeling of NF-κB regulation.

**Figure 4:**
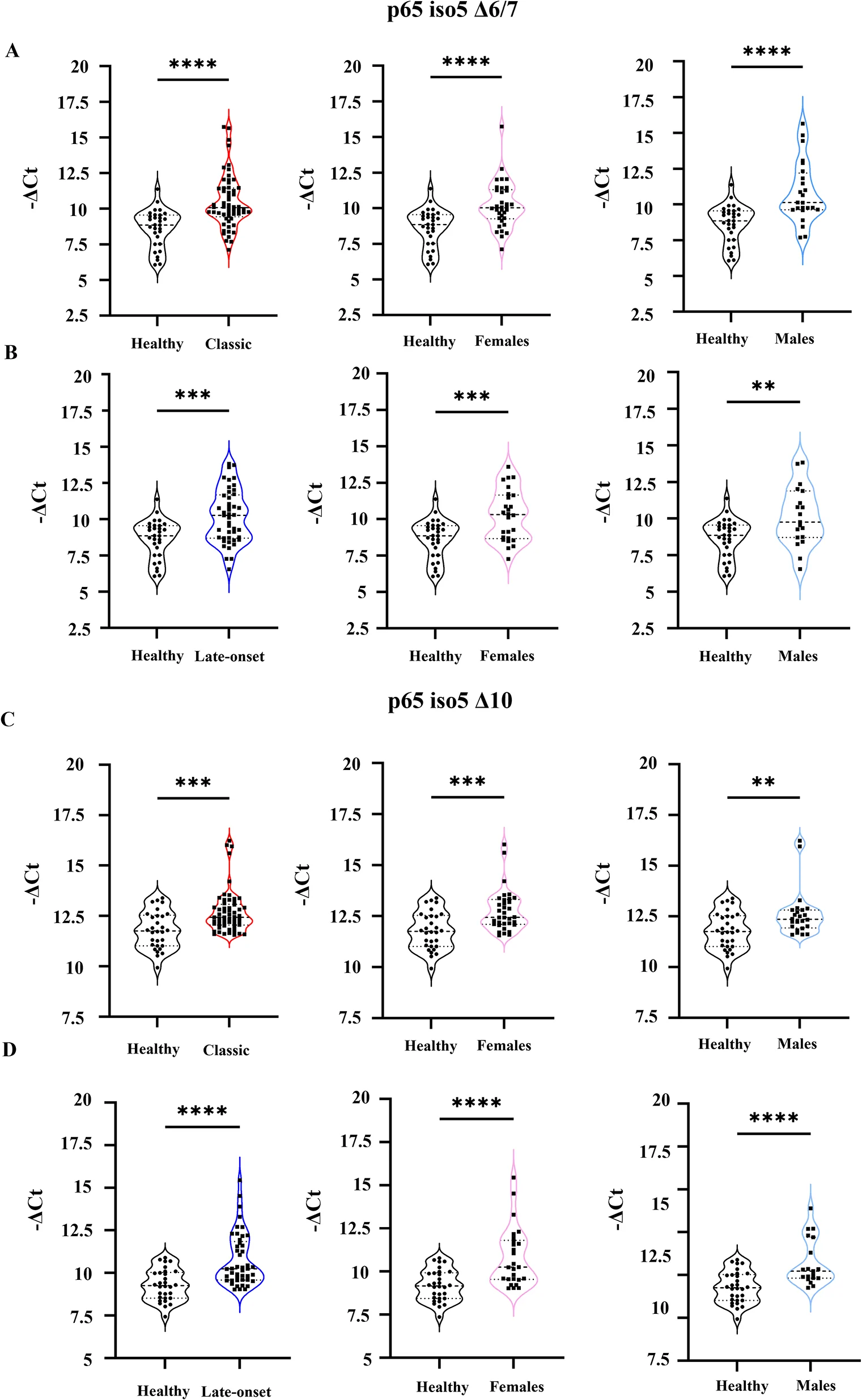
Differential mRNA expression of p65 iso5 Δ6/7 and p65 iso5 Δ10 in classic and late-onset Fabry disease. Violin plots show relative mRNA expression (-ΔCt) of p65 iso5 Δ6/7 and p65 iso5 Δ10 in healthy controls and patients with classic or late-onset FD. Data are stratified by disease phenotype and sex. **(A)** p65 iso5 Δ6/7 expression in healthy controls versus patients with the classic phenotype, shown for the total cohort (left), females (middle), and males (right). **(B)** p65 iso5 Δ6/7 expression in healthy controls versus patients with the late-onset phenotype, shown for the total cohort (left), females (middle), and males (right). **(C)** p65 iso5 Δ10 expression in healthy controls versus patients with the classic phenotype, shown for the total cohort (left), females (middle), and males (right). **(D)** p65 iso5 Δ10 expression in healthy controls versus patients with the late-onset phenotype, shown for the total cohort (left), females (middle), and males (right). Statistical significance is indicated as follows: *p < 0.05, **p < 0.01, ***p < 0.001, ****p < 0.0001.

### 3.4 Protein expression of p65 iso5 Δ6/7 in PBMCs

To determine whether transcriptional alterations were reflected at the protein level, total protein extracts from PBMCs of male and female subjects were analyzed by Western blot using an antibody recognizing the C-terminal region of p65. This antibody detects canonical p65, p65 iso5, and the lower-molecular-weight band corresponding to p65 iso5 Δ6/7 (**Table 9**). Cos-1 cells transfected with p65 iso5 Δ6/7 served as positive controls for band specificity. Although canonical p65 and p65 iso5 were also detectable in the Western blots, densitometric analysis was specifically performed for p65 iso5 Δ6/7 and normalized to β-actin. FD patients exhibited significantly increased levels of the p65 iso5 Δ6/7 protein compared with healthy controls, a pattern observed in both sexes. Densitometric analysis confirmed increased p65 iso5 Δ6/7 protein abundance in both classic (**Figure 5A**) and late-onset patients (**Figure 5B**). Overall, the increased protein expression of p65 iso5 Δ6/7 was consistent with the increased mRNA expression observed in both FD phenotypic subgroups. This concordance supports the biological relevance of p65 iso5 Δ6/7 and its potential involvement in NF-κB signaling in FD, although larger studies are needed to further clarify its role in disease-related inflammation.

**Table 9:** Predicted protein length of p65 iso5 and its alternatively spliced variants.

Isoform	Predicted amino acid lengths
*p65 iso5*	520
*p65 iso5 Δ6/7*	414
*p65 iso5 Δ10*	417

The table reports the predicted amino acid length of canonical p65 iso5 and the alternatively spliced variants p65 iso5 Δ6/7 and p65 iso5 Δ10. Deletion of exons 6 and 7 or part of exon 10 results in shorter protein isoforms compared with canonical p65 iso5.

**Figure 5:**
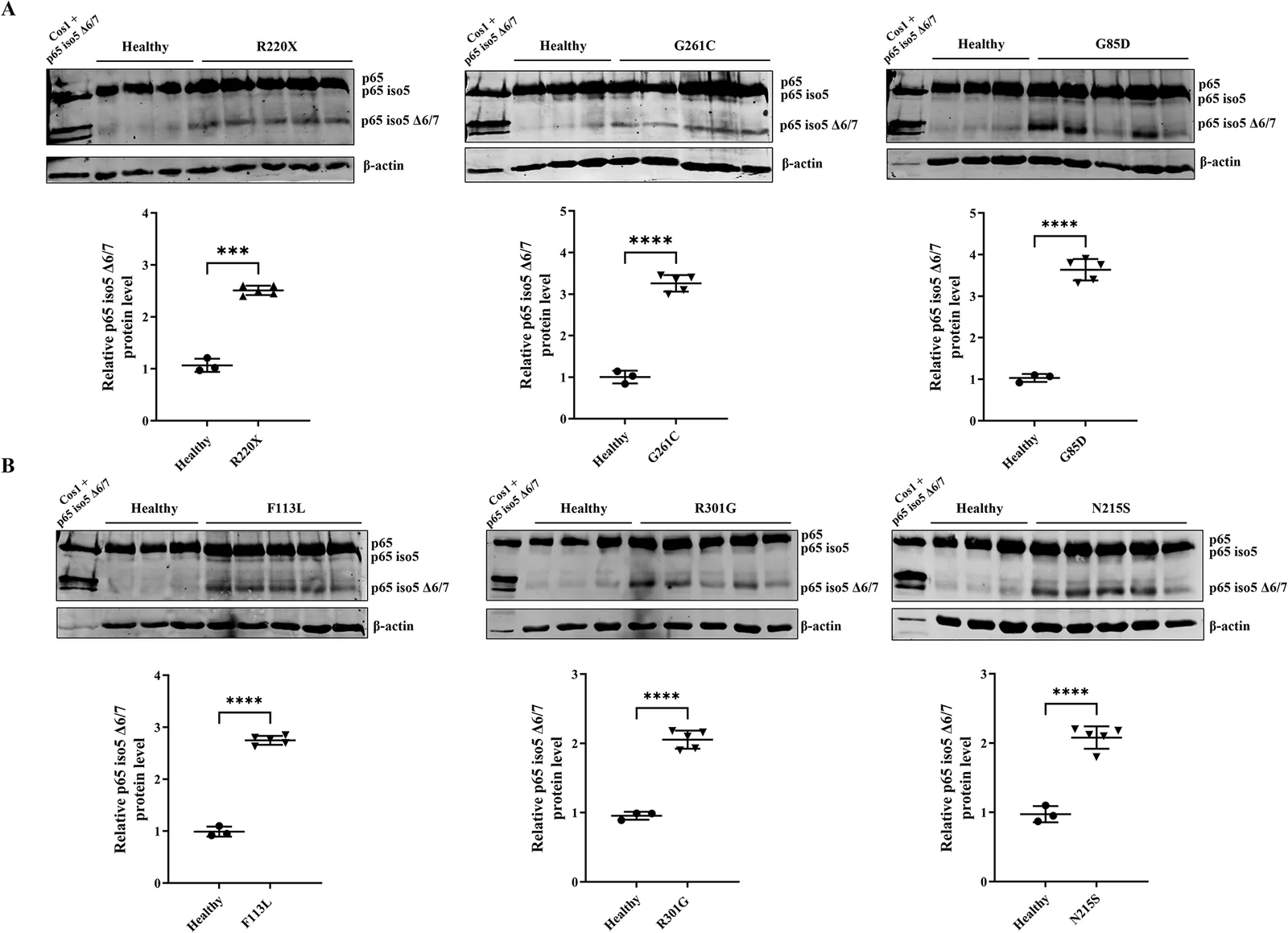
p65 iso5 Δ6/7 protein expression in PBMCs from patients carrying classic and late-onset *GLA* variants. **(A)** Western blot analysis and densitometric quantification of p65 iso5 Δ6/7 protein levels in PBMC protein extracts from patients carrying representative classic *GLA* variants (R220X, G261C, and G85D) and healthy controls. The Western blot shows canonical p65, p65 iso5, and p65 iso5 Δ6/7, as detected by the anti-p65 antibody. **(B)** Western blot analysis and densitometric quantification of p65 iso5 Δ6/7 protein levels in patients carrying representative late-onset *GLA* variants (F113L, R301G, and N215S) and healthy controls. Band intensities of the target protein and housekeeping protein were quantified by densitometric analysis and normalized considering the normalization factor. The level of protein expression is normalized with the mean of the healthy controls (*n* = 3), with each bar representing the standard deviation. Statistical significance is indicated as follows: **p < 0.01; ***p < 0.001; ****p < 0.0001.

### 3.5 mRNA and protein expression of p65 iso5 Δ6/7 and p65 iso5 Δ10 in PBMCs from GVUS subjects

Quantitative real-time PCR analysis revealed a significant upregulation of both splice variants in PBMCs from GVUS subjects relative to healthy controls (**Figures 6A, B**). Increased expression was evident in the overall cohort and remained consistent after stratification by sex, indicating that increased expression of these isoforms was observed in both males and females. To determine whether transcriptional changes were reflected at the protein level, p65 iso5 Δ6/7 expression was assessed by Western blot.

**Figure 6:**
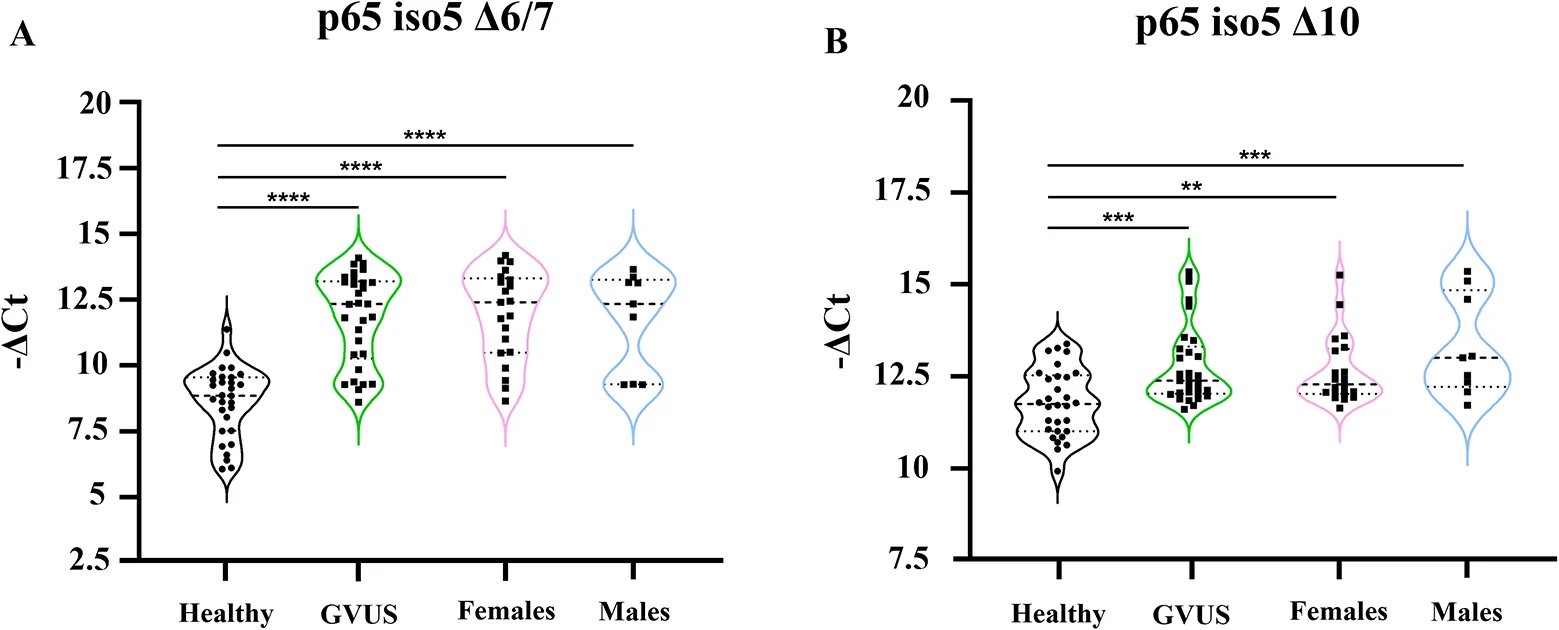
Increased p65 iso5 Δ6/7 and p65 iso5 Δ10 mRNA expression in subjects carrying *GLA* variants of uncertain significance. Violin plots show relative mRNA expression (-ΔCt) of **(A)** p65 iso5 Δ6/7 and **(B)** p65 iso5 Δ10 in healthy controls, the overall GVUS cohort, and GVUS female and male subgroups. The overall GVUS cohort includes both female and male subjects; therefore, the female and male subgroups are subsets of the overall GVUS cohort. Statistical significance was assessed by comparison with healthy controls. **p < 0.01, ***p < 0.001, ****p < 0.0001.

As shown in **Figures 7A, B**, protein quantification confirmed a significant increase in p65 iso5 Δ6/7 across different GVUS genotypes, including S126G, p.Gln107Arg (Q107R), and p.Met70Val (M70V). Although canonical p65 and p65 iso5 were also detectable in the Western blots, densitometric analysis was specifically performed for p65 iso5 Δ6/7 and normalized to β-actin. Results for A143T are reported in the following section. Overall, these findings show that increased expression of p65 iso5 Δ6/7 and p65 iso5 Δ10 occurs in GVUS subjects despite preserved α-GalA activity and normal Lyso-Gb3 levels.

**Figure 7:**
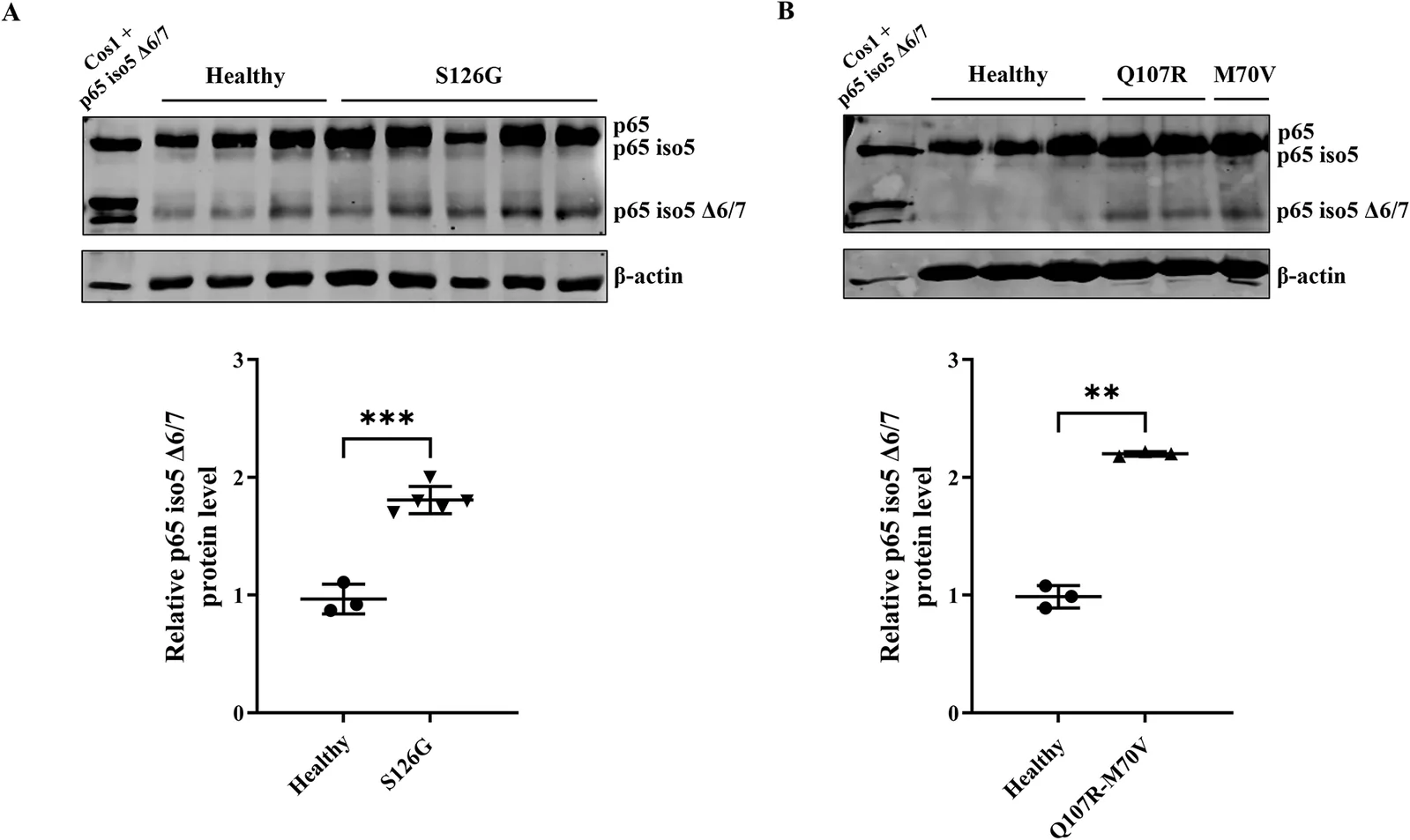
p65 iso5 Δ6/7 protein expression in PBMCs from subjects carrying GVUS *GLA* variants. **(A)** Western blot analysis and densitometric quantification of p65 iso5 Δ6/7 protein levels in PBMCs from healthy controls and subjects carrying the S126G variant. The Western blot shows canonical p65, p65 iso5, and p65 iso5 Δ6/7, as detected by the anti-p65 antibody. **(B)** Western blot analysis and densitometric quantification of p65 iso5 Δ6/7 protein levels in PBMCs from healthy controls and subjects carrying the Q107R and M70V variants. Band intensities of the target and housekeeping proteins were quantified by densitometric analysis and normalized considering the normalization factor. The level of protein expression is normalized with the mean of the healthy controls (*n* = 3), with each bar representing the standard deviation. Statistical significance is indicated as follows: **p < 0.01; ***p < 0.001; ****p < 0.0001.

### 3.6 Cytokine patterns and p65 iso5 Δ6/7 upregulation in A143T subjects

Particular attention was given to individuals carrying the A143T variant, a *GLA* missense mutation whose pathogenicity remains debated in the literature. Although many A143T subjects exhibit normal α-GalA activity and Lyso-Gb3 levels (50), several reports describe clinically relevant manifestations, including subclinical cerebrovascular involvement (51). This clinical-biochemical discrepancy makes A143T an informative model to investigate mechanisms beyond substrate accumulation. A143T subjects showed significantly increased p65 iso5 Δ6/7 transcript levels compared with healthy controls (**Figure 8A**). In addition, p65 iso5 Δ10 mRNA expression was significantly increased in A143T carriers compared with healthy controls, although the magnitude of this increase was less pronounced than that observed for p65 iso5 Δ6/7 (**Supplementary Figure S1**). Western blot analysis confirmed increased p65 iso5 Δ6/7 protein expression (**Figure 8B**), consistent with the observed transcriptional upregulation. Although canonical p65 and p65 iso5 were also detectable in the Western blot, densitometric analysis was specifically performed for p65 iso5 Δ6/7 and normalized to β-actin. To further characterize the inflammatory profile of A143T subjects, intracellular cytokine gene expression and circulating cytokine levels were analyzed. PBMCs from A143T subjects showed significantly increased TNF-α and IL-6 mRNA expression, together with increased IL-10 expression, whereas IL-4 expression remained unchanged (**Figures 8C, D**). Plasma cytokine profiling further revealed increased systemic levels of IFN-γ, IL-2, IL-8, IL-10, and IL-12 (**Figure 8E**). Overall, A143T subjects showed increased p65 iso5 Δ6/7 expression together with alterations in both intracellular cytokine expression and circulating cytokine levels, despite preserved α-GalA activity and normal Lyso-Gb3 levels. These findings identify a distinct inflammatory profile in A143T carriers that accompanies p65 iso5 Δ6/7 upregulation.

**Figure 8:**
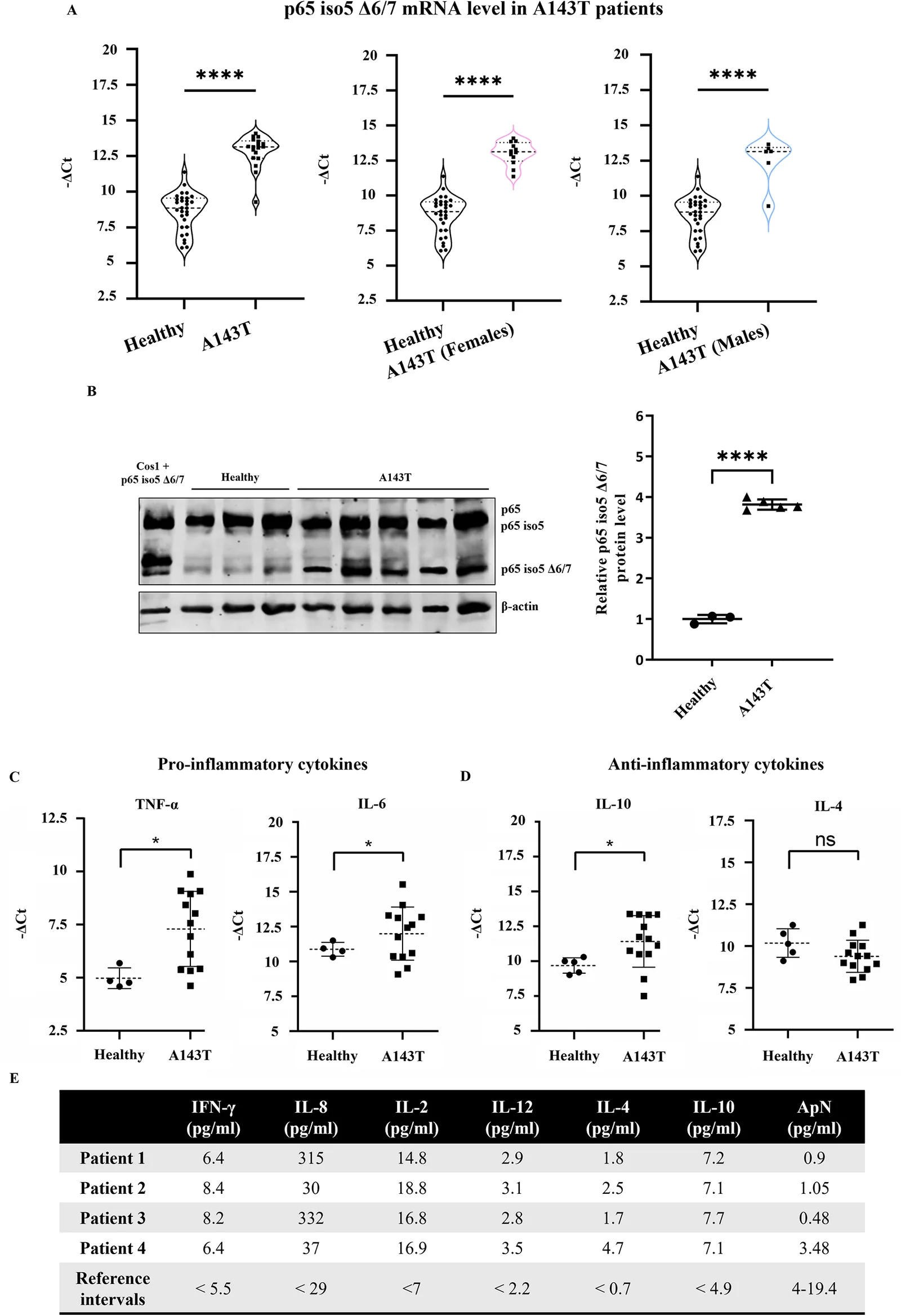
Upregulation of p65 iso5 Δ6/7 is associated with altered cytokine expression in individuals carrying the A143T *GLA* variant. **(A)** Relative mRNA expression of p65 iso5 Δ6/7 in PBMCs from healthy controls and individuals carrying the A143T variant. Data are expressed as -ΔCt values; ****p < 0.0001. **(B)** Western blot analysis and densitometric quantification of p65 iso5 Δ6/7 protein levels in PBMCs from healthy controls and A143T carriers. The Western blot shows canonical p65, p65 iso5, and p65 iso5 Δ6/7, as detected by the anti-p65 antibody. β-actin was used as the loading control. Band intensities of the target and housekeeping proteins were quantified by densitometric analysis and normalized using the corresponding normalization factor. The level of protein expression is normalized with the mean of the healthy controls (*n* = 3), with each bar representing the standard deviation. Statistical significance is indicated as follows: **p < 0.01; ***p < 0.001; ****p < 0.0001. **(C)** Relative mRNA expression of the pro-inflammatory cytokines TNF-α and IL-6 in PBMCs from healthy controls and A143T carriers. Data are expressed as -ΔCt values; * p < 0.05. **(D)** Relative mRNA expression of the anti-inflammatory cytokines IL-10 and IL-4 in PBMCs from healthy controls and A143T carriers. Data are expressed as -ΔCt values; * p < 0.05; ns, not significant. **(E)** Plasma cytokine concentrations in A143T carriers compared with the corresponding reference intervals. ApN, Adiponectin.

## 4 Discussion

Chronic inflammation is increasingly recognized as a key contributor to organ damage and disease progression in FD. This inflammatory component develops in parallel with lysosomal substrate accumulation, which is associated with oxidative stress, mitochondrial dysfunction, and activation of multiple inflammatory signaling pathways, including NF-κB (52). Although Gb3 and Lyso-Gb3 are known to stimulate pro-inflammatory cascades, including TLR4 and NF-κB-dependent signaling, the molecular mechanisms linking persistent lysosomal dysfunction to long-term immune dysregulation remain incompletely understood (1, 30). In the present study, we provide the first clinical characterization of the recently identified NF-κB splice variants p65 iso5 Δ6/7 and p65 iso5 Δ10 in Fabry disease. We demonstrate that both isoforms are significantly upregulated in patients with classic and late-onset FD, as well as in individuals carrying *GLA* variants of uncertain significance, identifying a common pattern of NF-κB splice variant redistribution across the Fabry disease spectrum. Together with the previously reported downregulation of p65 iso5, these findings suggest that chronic inflammatory stress in Fabry disease is associated with a selective remodeling of NF-κB splice variant expression rather than a uniform activation of the pathway. Alternative splicing is increasingly recognized as a mechanism shaping immune-cell responses and inflammatory signaling pathways. Similar patterns of disease-associated splice variant redistribution have been described in other chronic inflammatory conditions, supporting the concept that alternative splicing may represent an important mechanism involved in immune-inflammatory responses under persistent inflammatory stress (34–36). In this context, the selective upregulation of p65 iso5 Δ6/7 and p65 iso5 Δ10 observed in the present study suggests that NF-κB alternative splicing may constitute an additional regulatory layer contributing to immune-inflammatory remodeling in Fabry disease. Although NF-κB activation is a common feature of several inflammatory disorders, the divergent regulation observed across p65-derived splice variants suggests that disease-associated remodeling of NF-κB isoform composition may provide information beyond canonical pathway activation. Structurally, the two isoforms analyzed in this study harbor deletions affecting key functional domains of p65. The p65 iso5 Δ6/7 variant lacks a portion of the RHD, including regulatory residues involved in coactivator recruitment and glucocorticoid receptor-mediated trans-repression, whereas p65 iso5 Δ10 lacks part of the transactivation domain, potentially altering transcriptional efficiency and promoter selectivity. Despite these structural differences, both isoforms retain the ability to bind dexamethasone and translocate to the nucleus independently of IκBα, indicating engagement of noncanonical regulatory mechanisms (38) (39). These characteristics raise the possibility that changes in the relative abundance of individual p65 isoforms may influence the qualitative nature of NF-κB-dependent transcriptional responses and may be associated with the inflammatory phenotype observed in FD. However, the functional consequences of their increased expression in patient-derived cells remain to be established. Importantly, upregulation of these isoforms was not restricted to patients with marked enzymatic deficiency. Their increased expression in subjects carrying variants of uncertain significance, who typically exhibit preserved α-GalA activity and normal Lyso-Gb3 levels, indicates that NF-κB isoform remodeling may occur in the absence of detectable abnormalities in conventional biochemical markers. This observation is particularly relevant for understanding the clinical heterogeneity of GVUS subjects, who may present FD-like symptoms despite preserved biochemical parameters. In this context, altered inflammatory regulation may provide complementary information beyond conventional biochemical markers. Such signatures could be particularly informative in individuals carrying GVUS, where the relationship between genotype, biochemical findings, and clinical manifestations often remains difficult to interpret. Nevertheless, the present findings do not establish that altered NF-κB isoform expression is responsible for these clinical manifestations or determine the pathogenicity of individual *GLA* variants. Among GVUS, particular attention was given to the A143T variant, whose pathogenicity remains controversial. In our cohort, individuals carrying the A143T displayed marked upregulation of p65 iso5 Δ6/7 at both mRNA and protein levels, accompanied by increased expression of pro-inflammatory cytokines such as TNF-α and IL-6, as well as altered circulating cytokine levels. The concomitant increase in IL-10 may reflect activation of compensatory regulatory mechanisms, suggesting a complex remodeling of immune networks rather than a purely pro-inflammatory shift. Although these findings do not establish pathogenicity, they indicate that A143T carriers may exhibit measurable immune-inflammatory alterations despite the absence of the classical biochemical abnormalities typically associated with Fabry disease. The association between p65 iso5 Δ6/7 upregulation and altered cytokine profiles further supports the possibility that NF-κB splice variant remodeling may contribute to the molecular heterogeneity underlying the discordance between genotype, biochemical profile, and clinical phenotype observed in some A143T carriers. However, the present study does not establish a direct causal relationship between p65 iso5 Δ6/7 expression and cytokine production. The analysis was performed in PBMCs and was cross-sectional, and therefore further studies in disease-relevant tissues and longitudinal cohorts will be useful to further define the significance of the observed isoform changes. NF-κB isoforms expression may provide complementary molecular information in individuals with GVUS. Further functional studies will also help clarify the biological relevance and potential clinical utility of these NF-κB isoforms. In conclusion, this study provides the first clinical evidence that the NF-κB splice variants p65 iso5 Δ6/7 and p65 iso5 Δ10 are significantly upregulated in patients with classic and late-onset Fabry disease and in individuals carrying *GLA* variants of uncertain significance. The presence of these alterations in subjects with preserved α-GalA activity and normal Lyso-Gb3 levels suggests that NF-κB isoform remodeling may provide molecular information complementary to conventional biochemical markers, particularly in the context of unresolved genotype-phenotype relationships. The concomitant upregulation of p65 iso5 Δ6/7 and altered cytokine profiles observed in A143T carriers further supports an association between NF-κB alternative splicing and immune-inflammatory alterations, although these findings do not establish causality or variant pathogenicity. Together with the previously reported downregulation of p65 iso5, our findings support a model in which selective redistribution of NF-κB splice variants is associated with the molecular heterogeneity of inflammatory responses in Fabry disease. Further functional, longitudinal, and multicenter studies are warranted to clarify the biological significance and potential clinical utility of these isoforms.

## Data Availability

The sequences of p65 iso5 Δ6/7 (accession number PV942388) and p65 iso5 Δ10 (accession number PV942389) have been deposited in the NCBI GenBank database.
