# Supplementary material for: NF-κB p65 iso5 Δ6/7 and p65 iso5 Δ10 isoforms drive immune-inflammatory remodeling in Fabry disease and GLA variants of uncertain significance
Source: Front Immunol. 2026 Oct 6;17:1922658. doi: 10.3389/fimmu.2026.1922658 (PMC13680232; doi:10.3389/fimmu.2026.1922658)
Supplement: Supplementary file 1 [file Supplementaryfile1.docx]

Supplementary Material

# Supplementary Figures

**Supplementary Figure 1.** Increased p65 iso5 Δ10 mRNA expression in subjects carrying the A143T *GLA* variant. Violin plots show relative mRNA expression (-ΔCt) of p65 iso5 Δ10 in A143T subjects compared with healthy controls in the overall cohort (**A**), in female subjects (**B**), and in male subjects (**C**). Data are presented for the overall A143T subjects and stratified by sex. The same healthy control subjects were used as the reference group in the overall and sex-stratified comparisons; therefore, the corresponding control data points are repeated across the panels. Statistical significance is indicated as follows: *p < 0.05; **p < 0.01; ***p < 0.001; ****p < 0.0001.
